# Early biomarkers of joint damage in rheumatoid and psoriatic arthritis

**DOI:** 10.1186/s13075-015-0652-z

**Published:** 2015-06-01

**Authors:** Angela Mc Ardle, Brian Flatley, Stephen R. Pennington, Oliver FitzGerald

**Affiliations:** Conway Institute of Biomedical Research, University College Dublin, Belfield, Dublin 4 Ireland; Department of Rheumatology, St Vincent’s University Hospital, Elm Park, Dublin 4 Ireland

## Abstract

Joint destruction, as evidenced by radiographic findings, is a significant problem for patients suffering from rheumatoid arthritis and psoriatic arthritis. Inherently irreversible and frequently progressive, the process of joint damage begins at and even before the clinical onset of disease. However, rheumatoid and psoriatic arthropathies are heterogeneous in nature and not all patients progress to joint damage. It is therefore important to identify patients susceptible to joint destruction in order to initiate more aggressive treatment as soon as possible and thereby potentially prevent irreversible joint damage. At the same time, the high cost and potential side effects associated with aggressive treatment mean it is also important not to over treat patients and especially those who, even if left untreated, would not progress to joint destruction. It is therefore clear that a protein biomarker signature that could predict joint damage at an early stage would support more informed clinical decisions on the most appropriate treatment regimens for individual patients. Although many candidate biomarkers for rheumatoid and psoriatic arthritis have been reported in the literature, relatively few have reached clinical use and as a consequence the number of prognostic biomarkers used in rheumatology has remained relatively static for several years. It has become evident that a significant challenge in the transition of biomarker candidates to clinical diagnostic assays lies in the development of suitably robust biomarker assays, especially multiplexed assays, and their clinical validation in appropriate patient sample cohorts. Recent developments in mass spectrometry-based targeted quantitative protein measurements have transformed our ability to rapidly develop multiplexed protein biomarker assays. These advances are likely to have a significant impact on the validation of biomarkers in the future. In this review, we have comprehensively compiled a list of candidate biomarkers in rheumatoid and psoriatic arthritis, evaluated the evidence for their potential as biomarkers of bone (joint) damage, and outlined how mass spectrometry-based targeted and multiplexed measurement of candidate biomarker proteins is likely to accelerate their clinical validation and the development of clinical diagnostic tests.

## Introduction

Rheumatoid arthritis (RA) and psoriatic arthritis (PsA) are the most prevalent forms of inflammatory arthritis affecting ~1 % and ~ 0.3 to 1 % of the population, respectively [1, 2]. Disease aetiology is unknown but it is thought that both genetic and environmental factors trigger the onset of these arthropathies [3]. The onset of RA and PsA is clinically recognised when a patient presents with symptoms fulfilling disease classification criteria, importantly the American College of Rheumatology criteria for RA and the Classification for Psoriatic Arthritis CASPAR criteria [4, 5]. However, it is recognised that disease onset may occur much earlier, even prior to symptom onset [6, 7]. Several disease-specific characteristics differentiate RA from PsA. For example, rheumatoid factor (RF) is present often at high titre in 80 % of RA patients whereas it is present at low titre in only 13 % of PsA patients. PsA is included among the spondylarthopathies because it shares both clinical features and association with HLAB27 with other spondylarthopathy members. The presence of psoriasis is a hallmark of PsA, although joint involvement may precede skin manifestations in ~10 % of patients. Asymmetric joint involvement is seen commonly in PsA whereas joint involvement in RA follows a symmetrical pattern. Dactylitis, enthesitis, sacroiliitis and interphalangeal joint involvement are also more common in PsA [3].

**Fig. 1:**
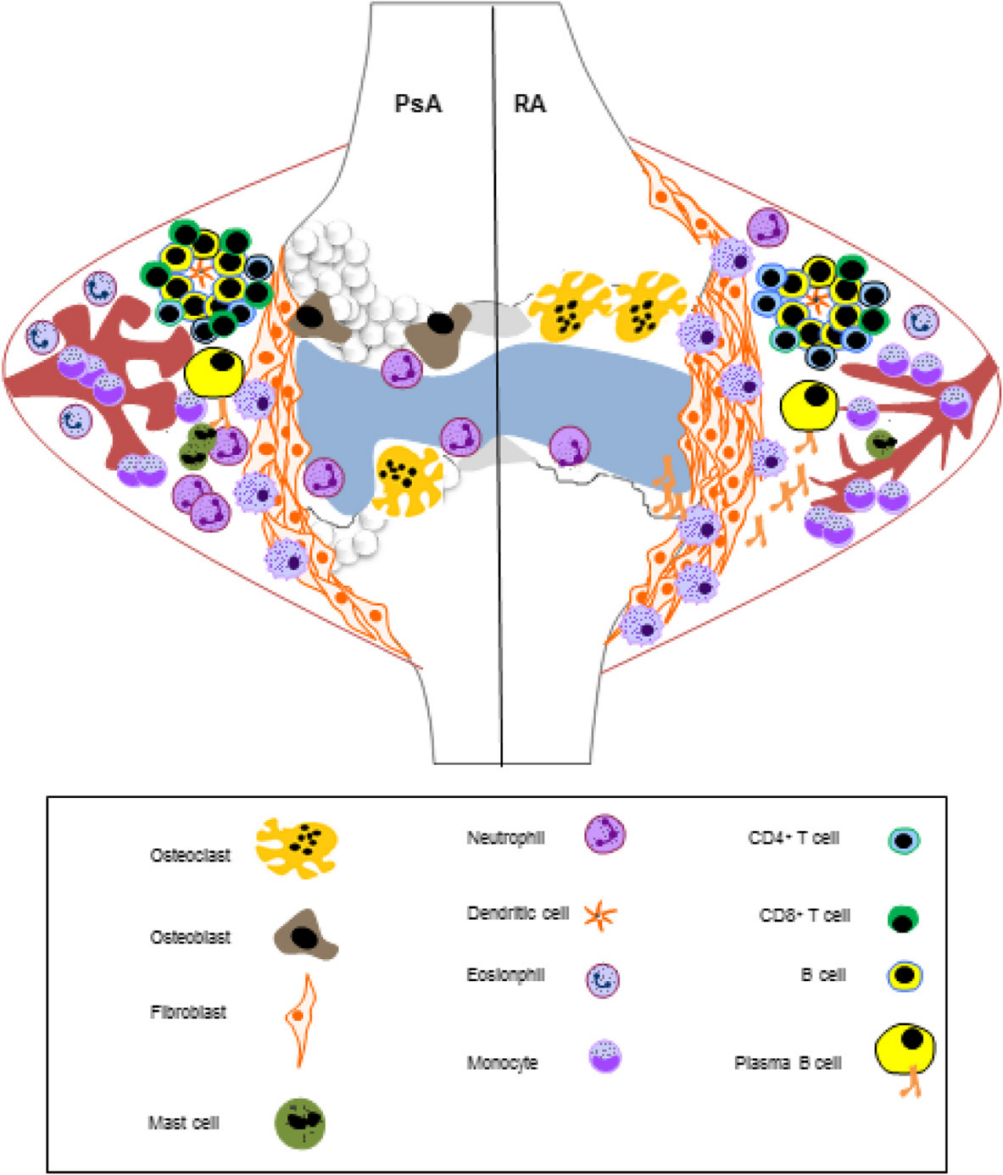
Synovitis in rheumatoid arthritis and psoriatic arthritis. Synovitis in rheumatoid arthritis (RA) and psoriatic arthritis (PsA) is triggered by unknown event(s). It is thought that a genetic predisposition and/or environmental cues trigger inappropriate activation of synoviocytes, thereby promoting an autoimmune inflammatory response. Once activated, synoviocytes produce proinflammatory cytokines that in turn activate proximal cells, including endothelial cells that line the blood vessels supplying the joint. This results in dysregulated angiogenesis and the increased infiltration of leukocytes, including monocytes, macrophages, neutrophils, mast cells, eosinophils, B cells and T cells. Infiltrating cells produce cytokines that act in synergy to propagate the inflammatory response. Importantly, tumour necrosis factor alpha (TNFα) and interleukin (IL)-17 are cytokines with major implied roles in PsA and RA pathogenesis and represent important therapeutic targets. With the development of a chronic inflammatory response, the synovial lining becomes hyperplastic. Fibroblasts and macrophages form an invasive matrix (pannus) that promotes the destruction of cartilage and bone. Activation of osteoclast cells promotes bone resorption whereas activation of osteoblasts promotes bone proliferation

At the cellular level, histological studies have revealed important differences between synovial tissue in RA and PsA [8]. Angiogenesis is dysregulated in both conditions and abnormal vessel morphology and function has been reported. Increased straight, branching vascularisation is a prominent feature observed in RA joints, whereas the formation of elongated, bushy, torturous blood vessels is a more marked feature of the PsA joint [8, 9]. In the RA joint there is increased macrophage infiltration and subsequent synovial invasion compared with that observed in PsA. As a result, lining layer hyperplasia observed in RA is more striking than that observed in PsA [3]. Conversely, PsA is characterised by more extensive infiltration of polymorphonuclear cells [8]. It has been reported that the extent of T-cell and B-cell infiltration is comparable in both conditions and the formation of germinal centres (zones of T-cell and B-cell proliferation) are observed in both PsA and RA joints [8, 10, 11]. The differences in synovitis in RA and PsA are illustrated in Fig. 1.

## Radiographic progression

Radiographic progression is considered a consequence of synovial inflammation. However, at least for RA, the observations that bone loss can occur before clinical onset and at very early stages of disease have been widely acknowledged [6, 7]. These observations are surprising since synovitis requires some time to destroy bone to an extent that is clinically detectable. Synovitis might thus not be the exclusive cause of joint damage. An alternative concept suggests that autoimmune processes begin years before the clinical onset of disease and that these processes promote joint destruction. Indeed, levels of anti-citrullinated protein antibodies (ACPA) can be detected in RA years before the clinical onset of disease. A comparative imaging study used micro computerised tomography to assess bone densities in healthy individuals that were either positive or negative for ACPA. The ACPA-positive individuals exhibited significant alterations in cortical bone architecture as compared with the ACPA-negative individuals. These findings support the theory that bone damage is not exclusively a consequence of synovitis [7].

**Fig. 2:**
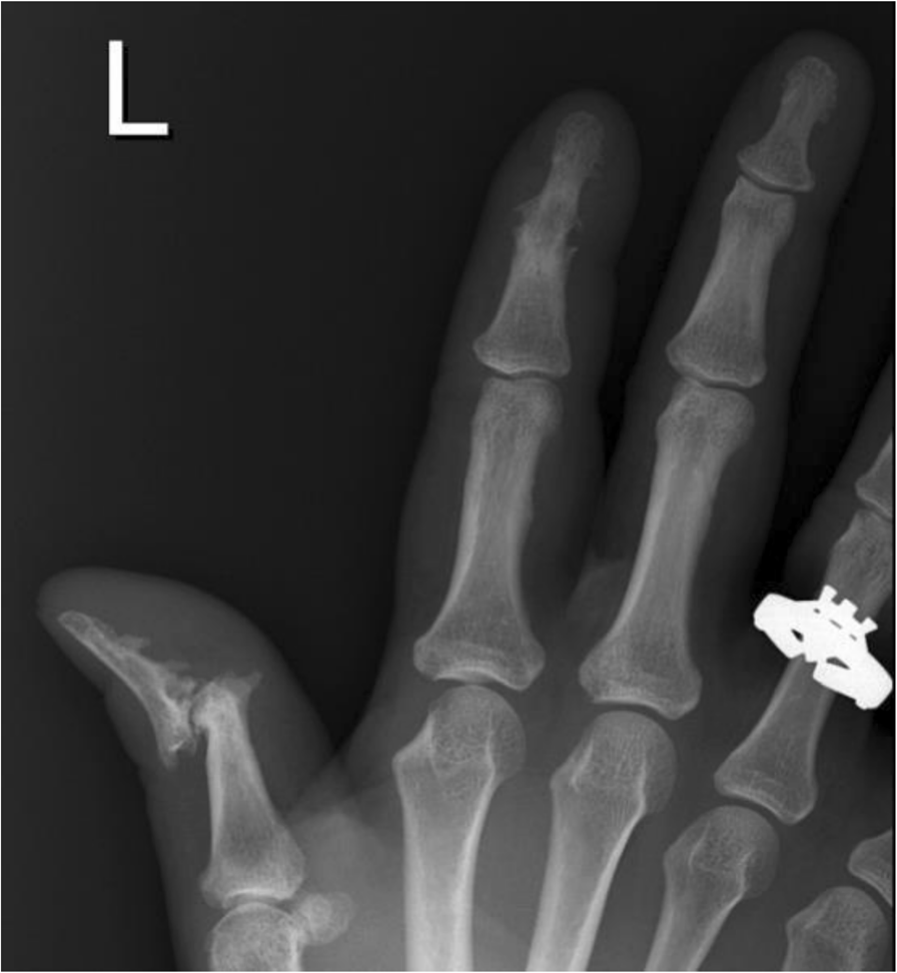
X-ray image of changes in bones observed in psoriatic arthritis. Bone changes in psoriatic arthritis (PsA) patients may differ between patients and may also differ within the same patient. The heterogeneity observed within a PsA patient is illustrated. Left-hand radiograph from a PsA patient showing severe erosive disease and subluxation at the first distal interphalangeal (DIP) with fluffy periosteal new bone formation on the terminal phalange. Ankylosis of the second DIP joint is also demonstrated

Previously, PsA was believed to be a mild nonprogressive form of arthritis. However, it is now well understood that 47 % of PsA patients will develop erosions within 2 years of symptom onset, and that of the patients suffering from polyarticular PsA at least 20 % are at risk of progressing to a severe destructive phenotype (mutilans) comparable with that observed in RA [12]. What is more, bone changes observed in PsA are particularly heterogeneous both between and also within individual sufferers (Fig. 2). X-ray images are used in the clinic to follow radiographic progression and may be scored to measure the extent of joint damage. The Sharp–van der Heijde scoring method is most commonly used to measure joint damage in RA. This method provides separate scores for erosion and joint space narrowing in the hands, wrists and feet. A modified Sharp–van der Heijde score is used to measure radiographic progression in PsA where the distal interphalangeal joints of the hands are also included [13, 14].

While X-ray imaging is the best validated technique for detecting bone erosion, it is limited by its two-dimensional character. Additional imaging modalities such as magnetic resonance imaging, micro computerised tomography and ultrasound are utilised to identify distinguishing features between RA and PsA. Radiographic progression in RA and PsA is remarkably different. RA is a bone-resorbing, erosive disease whereas the pattern of radiographic progression in PsA is more complex. As in RA, bone resorption and erosion may be evident in PsA, but radiographic progression may also be marked by more severe resorption or osteolysis and commonly bony proliferation is prominent. To the best of our knowledge early protein biomarkers of bone proliferation have not yet been reported in the literature, but these markers will probably differ from biomarkers of erosion. Distinguishing patterns of disease progression in RA and PsA have been captured by different imaging modalities and are summarised in Table 1.

**Table 1:** Radiological features that distinguish between rheumatoid arthritis and psoriatic arthritis

Disease feature	Rheumatoid arthritis	Psoriatic arthritis	Imaging technique	Reference
Number of erosions	+++	+	X-ray	[18]
	+++	+++	μCT	[82]
Severity of erosions	+++	++	μCT	[83]
Shape of erosions				
Ʊ	+	+++	μCT	[83]
Tubule	+	+++	μCT	
U	+++	+	μCT	
Erosion distribution	Preponderance for radial sites	Evenly distributed	μCT	[83]
DIP joint erosion	–	+++	US, MRI, X-ray	[84]
Number of osteophytes	+	+++	μCT	[82]
Severity of osteophytes (size)	+	+++	μCT	[82]
Bone proliferation	+	+++	US, MRI, X-ray	[84]
Inflammatory changes				
Synovitis	+++	++	MRI, US	[84]
Tenosynovitis	+++	++	MRI, US	[84]
Enthesitis	+	+++	MRI, US	[85]
Dactylitis	–	+++	US, MRI	[86]
Mutilans (erosions on both sides of joints)		+	X-ray	[87]

Disease features present in RA and PsA and the radiological imaging technique used to measure the feature. The number of erosions observed in RA appears to be greater than that in PsA. However, more sophisticated higher resolution techniques reveal this is not accurate because erosions in PsA are generally smaller and their detection requires these more sensitive techniques. Hence, μCT reveals a comparable extent of bone erosion in RA and PsA. MRI and US capture differences in sites affected by inflammation in these disorders. In PsA it is the enthesis that are major sites of inflammation, whereas in RA the synovium becomes chronically inflamed. Inflammation of the tendons is also prevalent in both disorders although more severe in RA. Distinct features of PsA include bony proliferation, and dactylitis. DIP, distal interphalangeal; μCT, micro computational tomography; MRI, magnetic resonance imaging; PsA, psoriatic arthritis; RA, rheumatoid arthritis; US, ultrasound

## Biomarkers

Molecular events that drive joint damage may precede disease onset and can cause detrimental long-term effects such as disability [6, 7, 10, 15, 16]. It is therefore essential to begin therapy as soon as possible in order to prevent irreversible damage. Some of the therapies currently available are associated with high cost and potential for side effects. Additionally, up to 40 % of patients will not meet the primary outcome measure and, in those who do respond, levels of disease activity can remain significant. To add to the complexity, not all patients will develop a destructive form of disease. Aggressive treatment should thus be reserved for patients that will develop a more severe form of disease [17]. In light of this, the need to develop biomarker signatures predictive of joint damage in RA and PsA is of critical importance. Currently there is a major international effort driven by the Outcome Measures in Rheumatology Clinical Trials and the Group for Research and Assessment in Psoriasis and Psoriatic Arthritis well underway in RA and to start soon in PsA whereby biomarker samples are being prospectively collected in an effort to identify predictors of radiographic damage.

Biomarkers serve as objective molecular indicators of pathological processes such as the development of joint destruction. Owing to the heterogeneous nature of RA and PsA, identifying a single biomarker predictive of joint damage would be an onerous task. Indeed, no single biomarker has so far emerged as a reliable predictor of damage in RA [18–20]. Consequently there is great interest in identifying a panel of biomarkers that could be incorporated into a signature predictive of disease progression. Such a signature could be used as an indicator of joint destruction and thus facilitate clinicians in making more informed clinical decisions and help guide personalised medical approaches [18, 20]. It is important to recognise that joint destruction contributes significantly to disability in patients suffering from inflammatory arthritis, but it is not the sole cause of disability. Joint inflammation, swelling, pain and disability occur in the absence of joint destruction [21]. In PsA, for example, there are many debilitating phenotypes of disease but not all PsA phenotypes include bone erosion [22]. It would be useful to have a biomarker that distinguishes between different disease phenotypes (for example, progressors from nonprogressors). Such a biomarker would help inform the clinical decision of whether to treat symptoms only or to adopt a more aggressive treatment strategy in order to prevent radiographic progression.

The aim of this review is to identify previously published reports that include proteins predictive of joint damage at early disease time points. Joint damage is most definitively measured by radiographic progression. Hence this review is limited to observational prospective cohort studies that used radiographic progression as a measure of joint destruction and included patients with early-stage disease at baseline. Observational studies are considered inferior to randomised control trials due to the fact that results can be influenced by confounding factors such as a patient’s response to therapy. Biomarkers predictive of joint damage at baseline in a treatment-naïve patient may not be predictive after initiation of therapy due to the suppressive effect of the pharmacological intervention on inflammatory mediators. However, evidence derived from well-designed prospective cohort studies that follow patients longitudinally and correct for confounding factors is considered important in the clinical decision-making process and is comparable with that provided by randomised control trials [23, 24]. Limiting this approach, there is a paucity of early cohort studies in PsA in which radiographic progression is measured. As disease activity and changes in tissue organisation may serve as surrogate measures of joint damage, we have included soluble biomarkers that correlated with these disease parameters in the PsA studies reviewed. In addition, the potential opportunities afforded by new mass spectrometry-based protein measurement approaches will be highlighted. These methods are reaching clinical diagnostics application and have the potential to change dramatically the landscape of protein biomarkers.

## Biomarkers of joint damage in rheumatoid arthritis

### Markers of the acute phase response

Inflammation in the synovium is reflected by a systemic inflammatory response. Increased erythrocyte sedimentation rate (ESR) and hepatocyte production of acute phase proteins including C-reactive protein (CRP) and acute phase serum amyloid A (A-SAA) are surrogate markers of this process. The acute phase response has been considered important in the context of RA as it mirrors synovial inflammation. CRP and the ESR have been shown to correlate with radiographic progression and these indices have been incorporated into composite scores typically used to predict damage. However, these are general markers of inflammation; they are not disease specific. Furthermore, CRP and the ESR remain normal in a proportion of RA patients that may progress – and thus additional and more sensitive predictors are required. One RA cohort study which incorporated patients with a range of disease durations showed that A-SAA correlated with radiographic progression. It would be interesting to investigate the correlation of A-SAA with joint damage in an early cohort as this protein reflects not only systemic but also local inflammation [25].

### Auto-antibodies

Auto-antibodies such as RF and ACPA are very useful prognostic markers in RA. It has previously been reported that patients positive for ACPA or RF are more likely to develop erosions compared with those who are negative for both. Additionally it was reported that those who were positive for ACPA and negative for RF were more likely to progress to a severe disease state compared with those who were RF-positive but ACPA-negative. Furthermore it was noted that patients positive for both ACPA and RF were at greatest risk of disease progression [26–28]. ACPA emerge as the most useful indicator of joint damage. Indeed, RF is quite limited in its ability to predict damage because this antibody is not disease specific. Moreover RF is considered useful only at early disease time points, and its predictive power is lost as disease progresses. In contrast, in addition to being more sensitive, ACPA are more disease specific and useful at both early and later disease time points [26, 29]. There exist, however, subgroups of patients who are susceptible to joint damage but who test negative for both RF and ACPA. Since RF and ACPA have been proven very useful indicators of damage, the identification of novel auto-antibodies may prove advantageous especially in patients who lack RF and ACPA. It was reported recently that a distinct family of auto-antibodies which recognise carbamylated antigens, the anti-carbamylated protein antibodies, are predictive of a severe disease course in early RA patients even after correction for RF and ACPA. It was demonstrated that patients who were positive for immunoglobulin G anti-carbamylated protein antibodies but negative for ACPA developed a more severe disease course compared with patients who were ACPA-positive only. Those who were positive for both anti-carbamylated protein antibodies and ACPA were comparable in severity [30]. Anti-carbamylated protein antibodies might thus prove a useful biomarker in RA, and their ability to predict joint damage warrants further research.

### Cytokines and chemokines

Cytokines play a major role in promoting joint damage and their association with radiographic progression has been investigated in several early RA cohort studies. It has been reported separately that elevated levels of interleukin (IL)-6, IL-16, IL-22, IL-33, chemokine ligand (CCL)11 and chemokine (C-X-C) motif ligand (CXCL)13 are predictive of radiographic progression [31–35]. The finding that IL-6 was associated with joint damage is controversial, however, and contradictory findings have been reported. Klein-Wieringa and colleagues demonstrated that elevated levels of IL-6 were not significantly correlated with radiographic progression in an early RA cohort [36]. This is in disagreement with Knudsen and colleagues, who demonstrated that IL-6 was a strong independent predictor of radiographic progression [31]. These controversial findings might be explained in part by differences in study design. Klein-Wieringa and colleagues measured serum levels of IL-6 in a large RA cohort consisting of 253 patients. Patients enrolled in this study received three different treatment strategies. Radiographic progression was assessed over 4 years using the Sharp–van der Heijde scoring method. Multivariate regression analysis was used to find an association between levels of IL-6 and disease progression. This model corrected for confounding factors including therapeutic intervention. In contrast, Knudsen and colleagues measured plasma levels of IL-6 in a smaller RA cohort consisting of 51 patients and treatment change was permitted over the duration of the study. Radiographic progression was measured using the Larsen method. Levels of IL-6 were measured 13 times over 2 years and the mean concentration (area under the curve) of IL-6 measurements over 24 months were used to associate levels of IL-6 with bone erosions observed at 12 and 24 months [31, 36]. Conversely, a study by van Leeuwen and colleagues demonstrated that elevated levels of plasma IL-6 were not predictive of damage [37]. However, the IL-6 assay used during this study was less sensitive than that used by Knudsen and colleagues [31]. The differential findings in relation to IL-6 highlight the impact of study design on reported findings in the literature. Single reports provide evidence that IL-16, IL-22, IL-33, CCL11 and CXCL13 retain prognostic capacity in early RA. It was interesting to note that CCL11 was associated with reduced radiographic progression and thus may have a protective role during pathogenesis [33]. Additional studies should be carried out to validate the finding that the aforementioned molecules independently correlate with radiographic progression.

### Adipokines

Adipokines are cytokines produced by fat cells. These proteins are elevated in patients with RA, where they are thought to have potent immunomodulatory effects. A study by Rho and colleagues demonstrated that adiponectin, visfatin and leptin correlated with measures of radiographic progression. Adiponectin and visfatin were shown to correlate with increased radiographic progression, whereas leptin was associated with reduced progression [38]. Klein-Wieringa and colleagues confirmed the finding that adiponectin is predictive of radiographic progression in patients with early RA [36].

### Calprotectin

Calprotectin is released from activated leukocytes that derive mainly from the inflamed synovium in RA patients. A recent cohort study by Hammer and colleagues demonstrated that RA patients with higher baseline levels of calprotectin developed more severe radiographic damage after 10 years compared with patients with lower levels of calprotectin. Furthermore, calprotectin levels measured at baseline and at 10 years significantly correlated with radiographic damage after 10 years. The correlation observed for calprotectin was similar to that observed for CRP and ESR [39]. Taken together these results can lead to the conclusion that calprotectin is more specific and just as sensitive at predicting joint damage as CRP and may provide valuable information in the context of a biomarker signature predictive of joint damage.

### Markers of angiogenesis

Angiogenesis is central to the development of RA synovitis and thus assessing levels of angiogenic markers in RA patients may prove useful for predicting joint damage. Vascular endothelial growth factor (VEGF) is thought to be the most important mediator of angiogenesis and there is good evidence to suggest that this molecule should be considered a candidate marker of joint damage in RA [40, 41]. A study by Ballara and colleagues demonstrated in early RA that elevated VEGF levels at inclusion significantly correlated with radiographic progression after 1 year [41]. Clavel and colleagues confirmed the finding that VEGF was predictive of joint damage in early RA patients. Additional analysis revealed that VEGF levels at inclusion were correlated with initial Sharp scores and Sharp scores measured after 1 year. It was also demonstrated that levels of angiopoeitin-1 (a marker of angiogenesis) were predictive of damage at inclusion and after 1 year. This finding suggests that VEGF and angiopotietin-1 remain predicative at later disease stages after initiation of therapy [40].

### Products of collagen degradation

Fragments released as a result of type I and type II collagen degradation – including C-terminal telopeptide of collagen (CTX)I and CTXII, collagen type II degradation product epitopes C2C and C1,2C as well as matrix proteins such as cartilage oligomatrix protein (COMP) – are reflective of bone and cartilage damage. Several studies have investigated the association of these molecules with radiographic progression in early RA patients. Three authors reported that elevated levels of CTXI and CTXII are associated with long-term radiographic progression in early RA patients [42–44]. What is more, Garnero and colleagues reported that CTXI and CTXII were even more predictive of joint damage compared with CRP and ESR [42]. Bakker and colleagues found that elevated baseline levels of C1,2C were associated with radiographic progression after 1 year of treatment [45]. Additionally Verstappen and colleagues demonstrated that C2C significantly correlated with radiographic progression after 1 year and importantly remained predictive the following year [46]. Andersson and colleagues demonstrated that increments in COMP levels during a 3-month period following diagnosis were predictive of joint damage at 1-year, 2-year and 5-year follow-up [47]. The culmination of positive findings makes these degradation products attractive candidates for a biomarker panel predictive of joint damage.

### Enzyme mediators of destruction

The association of matrix metalloproteinase (MMPs), proteases that promote cartilage breakdown, with joint damage has also been investigated. Previous early RA cohort studies demonstrated that elevated levels of MMP-3 at baseline correlate significantly with radiographic progression [48–50]. However, another study documented that serial, longitudinal measurements of MMP-3 fail to correlate with measures of joint damage [51]. It can thus be speculated that MMP-3 may be a useful predictive marker of joint destruction at disease onset prior to treatment. MMP-1 might also have prognostic utility and its association with joint damage has been assessed. Previously, elevated baseline levels of MMP-1 were demonstrated to significantly correlate with radiographic progression observed at 12 months [49]. It has also been reported that serial measurements of MMP-1 over a period of 18 months correlate significantly with measures of joint damage [51]. In contrast, Young-Min and colleagues found no correlation between levels of MMP-1 and joint damage in early RA patients [50]. It is certainly possible that differences in study design and data analysis gave rise to these discrepancies, and the relationship between MMP-1 and joint damage warrants further investigation.

## Biomarkers of joint damage in psoriatic arthritis

### Cytokines

The cytokine expression profile in the PsA synovium is relatively similar to that observed in RA [8]. IL-17 has emerged as an important cytokine in autoimmune diseases and this protein in synergy with tumour necrosis factor alpha contributes to pathogenesis in RA and PsA. It has previously been demonstrated that, compared with osteoarthritic controls, levels of IL-17 are elevated in the synovial tissue of patients with PsA and RA (no significant difference between the two). It was also demonstrated that in vitro stimulation of synovial tissue with IL-17 induced proteins involved in matrix turnover and cartilage destruction [52].

There are very few studies reporting the association of soluble cytokines with joint damage in PsA. However, cytokines that segregate patients with polyarthritis from those with oligoarticular disease have been identified. In an early PsA cohort, elevated levels of IL-1 were detected in the synovial fluid of polyarthritic patients compared with those with monoarthritis, suggesting that this protein is a marker of disease progression [53]. A Norwegian cohort found that elevated levels of IL-12p40, interferon alpha, IL-15 and CCL3 could segregate PsA patients with polyarticular disease from those with oligoarticular disease [54]. Since patients with polyarthritis have more severe joint involvement, these molecules might drive progression and would be interesting to investigate in the context of early PsA.

### Calgranulin (S100A8/S100A9)

A study by Kane and colleagues demonstrated that elevated levels of calgranulin (S100A8/S100A9) correlated with measures of disease activity and markers of intra-articular inflammation (white blood cell counts). The study further demonstrated that treatment with methotrexate resulted in a significant decrease in S100A8/S100A9 levels. A significant reduction in swollen joint count, Richie articular index and Disease Activity Score was also observed after treatment, suggesting that S100A8/S100A9 may also correlate with joint damage in PsA [55]. Additional evidence reporting an association between S100A8/S100A9 with progressive disease exists. A study by Aochi and colleagues demonstrated that S100A8/S100A9 levels in PsA patients (disease duration not specified) with more than 10 affected joints were higher compared with levels in those who had less than 10 affected joints [56]. These results together suggest that S100A8/S100A9 may be associated with joint damage and that this marker might provide additional information in the context of a biomarker signature.

### Markers of angiogenesis

The ability of angiogenic markers to predict joint damage in RA has been reported [41, 57]. Abnormalities in angiogenesis are more pronounced in PsA compared with RA and levels of VEGF and angiopotietin-2 have been reported to be higher during PsA relative to RA [56]. It is thus logical to conclude that these molecules could act as early markers of radiographic progression, but there is a lack of data in the literature to validate this hypothesis. Interestingly, the association of VEGF with active versus inactive disease and with changes in synovial vascular morphology has been described [56–58].

### Molecules that regulate bone turnover

Dalbeth and colleagues examined the association between soluble mediators of bone remodelling (receptor activator of nuclear factor-κB ligand, osteoprotegerin, wnt signalling pathway inhibitor-1, macrophage colony-stimulating factor) with radiographic progression in PsA patients with established disease duration. A positive correlation between macrophage colony-stimulating factor and receptor activator of nuclear factor-κB ligand concentrations with radiographic progression was described [59]. Connolly and colleagues found that baseline levels of A-SAA were independently associated with 1-year radiographic progression in PsA patients with long disease duration. A-SAA promotes the production of MMPs by fibroblast-like synoviocytes. A-SAA levels were demonstrated to correlate with MMP-1, MMP-3, MMP-13 and MMP/tissue inhibitor of matrix metalloproteinases [25]. Since it has been demonstrated previously that MMP-1 and MMP-3 are associated with radiographic progression in early RA, it could be speculated that an association between A-SAA and MMPs might correlate with radiographic progression in early PsA – this warrants further research.

## An emerging mass spectrometry technology

The development of a protein biomarker identified in discovery experiments for clinical assay is both long and challenging [60]. The clinical validation of candidate biomarkers has traditionally relied on the development of antibody-based assays. Enzyme-linked immunosorbent assays (ELISAs) are one of the most widely used methods for clinical diagnostic protein biomarker measurements [61, 62].

Large numbers of candidate biomarkers are emerging and it has become apparent that an alternative technology is required to evaluate them in a time- and cost-effective manner [63, 64]. The relatively recent introduction of a multiple reaction monitoring (MRM) mass spectrometry platform for the measurement of peptides has provided the opportunity to develop multiplexed assays for simultaneously measuring multiple candidate biomarkers and to progress them through the biomarker development pipeline [65]. There are a number of key drivers for the adoption of MRM as a viable alternative to the traditional antibody-based approach. The economics of MRM analysis far outweigh methods requiring antibodies (western blot or ELISA). Good antibodies are relatively expensive and in some cases not always available for the proteins of interest. Even for proteins for which antibodies are available, the length of time needed to optimise an assay using MRM mass spectrometry is much shorter relative to that using antibody-based protein detection [63, 64].

MRM assays are developed for peptides released from proteolytically cleaved proteins. A peptide that is unique to the protein of interest (a proteotypic peptide, generally ranging from 7 to 25 amino acids in length) and that is routinely observed by the mass spectrometer is crucial to guaranteeing accurate detection of specific proteins [66]. By measuring only selected proteotypic peptides, the abundance of selected proteins can be definitively established [65, 67, 68]. In MRM mode, only those peptides of interest pass through the mass spectrometer by setting the first quadrupole to filter based on the known mass/charge ratio of the peptide. In the second quadrupole the peptides are then fragmented and will produce fragments of known size. The third quadrupole is then used to filter these fragments, allowing them to pass to a detector. The prior knowledge of peptide sequences is used to direct the mass spectrometry in MRM mode. The triple-quadrupole mass spectrometer can be set to filter hundreds of peptides in the first quadrupole and thousands of peptide fragments (transitions) in the third quadrupole, thereby enabling many proteins to be measured. Therefore, for example, in a typical analytical run (<30 minutes) it is possible to measure hundreds of peptides [69, 70], which is analogous to performing hundreds of western blot analyses or ELISAs within a 30-minute timeframe. Indeed the MRM approach has been implemented in several large-scale biomarker studies over the past couple of years. These assays have so far proved particularly useful in the field of toxicology and oncology [71–73]. This technique has not yet been exploited to its full potential in rheumatology, but evidence supporting its potential utility does exist. For example, an MRM assay was developed for CRP and this assay was then used to distinguish progressive from nonprogressive arthritic patients [74]. More recently, Ademowo and colleagues developed an MRM assay for a panel of 57 synovial tissue proteins. This assay was then used to predict PsA patients who responded well to therapy compared with those who did not respond [75].

Luminex technology is another example of a multiplex platform for biomarker validation. This technology is based on polystyrene beads that are coated with specific capture antibodies and impregnated with dyes of different intensities. Interrogation of the beads with lasers results in the identification of a bead and hence an analyte due to its unique spectral properties [76]. The luminex is cable of measuring proteins that span a low dynamic range (<10^3^) which would not be detected by MRM. In addition to being a high-throughput assay, the sensitivity and specificity of the luminex technique is comparable with the ELISA [77, 78]. This technology is limited, however, by availability of antibodies against proteins of interest, high cost and the quantity of sample required for analysis [78].

## Conclusions

In relation to what has been shown to date, considerably more candidate biomarkers have been identified in RA compared with PsA (Table 2). No single biomarker has been validated as a potent predictor of joint damage and it is well recognised that a multi-biomarker panel is needed to compensate for the heterogeneity between individuals. A multi-biomarker panel incorporating 12 serum proteins has been shown to accurately reflect disease activity in RA [18]. This panel of proteins may be measured using a blood-based test referred to as a multi-biomarker disease activity (MBDA) test. Concentrations of the 12 biomarkers are incorporated into an algorithm that provides a low, moderate or high disease activity score as an output [18]. A recent study has shown that the MBDA test correlates well with measures of CRP, but there may also be discordance between levels of CRP and the MBDA test. In patients with both low levels of CRP and a low MBDA score, radiographic progression is infrequent. In contrast, in patients with low levels of CRP but a high MBDA score, a significant proportion developed radiographic progression during 1 year of follow-up. Levels of the multi-biomarker panel (which include CRP) thus better predict radiographic progression than CRP alone [79]. In a further study the MBDA test has been shown to predict risk of radiographic progression and outperform CRP as a predictive biomarker in the SWEFOT cohort trial. In this trial, only patients with a high MBDA score were at risk of developing radiographic progression. In contrast, a substantial proportion of patients with low, moderate and high levels of CRP were at risk of developing joint damage [80]. Finally, the ability of the MBDA score to predict joint damage has been demonstrated in an additional trial that included 163 patients from the Leiden Early Arthritis Cohort. The MBDA score and the Disease Activity Score in 28 joints–CRP score were assessed as predictors of radiographic progression. The study found that patients with low MBDA had significantly less radiographic progression than patients who meet Disease Activity Score in 28 joints–CRP European League Against Rheumatism defined remission. It was also shown that patients with a high MBDA score were six times more likely to develop radiographic progression compared with patients with a high Disease Activity Score in 28 joints–CRP score, who were only twice as likely to progress [81].

**Table 2:** Candidate biomarkers of joint damage in rheumatoid arthritis and psoriatic arthritis

Candidate biomarker	Evidence for role in inflammatory arthritis	Use
Inflammatory proteins		
C-reactive protein	Opsonisation and compliment activation	RA
Calprotectin (S100A12)	Ca^2+^ binding protein released upon phagocyte activation, important intracellular and extracellular roles	RA/PsA
Calgranulin (S100A8/S100A9)	Ca^2+^ binding protein with pleotropic effects. Regulates myeloid derived cells	PsA
A-SAA	Promotes the production of MMPs	RA/PsA
Cytokines		
IL-1	Promotes activation of keratocytes, endothelial cells, chondrocytes and osteoclasts. Promotes the production of proinflammatory cytokines	PsA
IL-6	Promotes neutrophil chemotaxis and production of proinflammatory cytokines, induces an acute phase response	RA
IL-13	Promotes antibody production by B cells	RA
IL-15	Induces T cell proliferation and B cell differentiation. Recruits memory T cells to the synovium and induces TNFα production	PsA
IL-16	Promotes chemotaxis of CD4^+^ T cells, monocytes and eosinophils. Modulates T-cell activation	RA
IL-22	Induces proliferation of fibroblasts and production of MCP-1 (monocyte chemokine)	RA
IL-33	Promotes chronic inflammatory response	RA
Chemokines		
CCL3	Lymphocyte, monocyte, basophil, eosinophil chemoattractant	PsA
CCL11	Eosinophil chemoattractant	PsA
CXCL13	B-cell chemoattractant	RA
Adipokines		
Adiponectin	Induces IL-6 and MMP-1 production by SLFs. Promotes IL-6, TNFα and MCP-1 production in chondrocytes	RA
Visfatin	Role unclear, thought to modulate inflammation	RA
Markers of angiogenesis		
VEGF	Potent inducer of angiogenesis and vascular permeability	RA/PsA
Angiopotietin-1	Promotes angiogenesis (growth of new blood vessels)	RA
Angiopotietin-2	Promotes angiogenesis	PsA
Auto-antibodies		
Rheumatoid factor	Forms immune complexes, promotes complement activation and formation of rheumatoid nodules	RA
Anti-CCP	Promotes complement activation	RA
Anti-Carp	Bind homocitrulline containing proteins	RA
Enzyme mediators of destruction		
MMP-1	Degrades collagen	RA
MMP-3	Degrades collagen	RA
Regulators of bone remodelling		
RANKL	Induces osteoclast bone destruction	PsA
M-CSF	Induces aggressive phenotype in macrophages	PsA
Products of collagen degradation		
COMP	Cartilage oligomatrix protein	RA
CTXI	C-terminal telopeptide of collagen type I	RA
CTXII	C-terminal telopeptide of collagen type I	RA
C1,2C	Collagen type II degradation product	RA
C2C	Collagen type II degradation product	RA

Candidate biomarkers predictive of joint damage in RA and PsA have been identified in the literature. These include inflammatory proteins, cytokines, chemokines, adipokines, markers of angiogenesis, auto-antibodies, enzyme mediators of destruction, molecules that regulate bone turnover and products of collagen degradation. For references see text. A-SAA acute-phase serum amyloid A; anti-Carp, anti-carbamylated protein antibodies; CCL, chemokine ligand; CCP, cyclic citrullinated peptide; CXCL, chemokine (C-X-C) motif ligand; IL, interleukin; M-CSF, macrophage colony stimulating factor; MCP-1, monocyte chemoattractant protein-1; MMP, matrix metalloproteinase; RA, rheumatoid arthritis; PsA, psoriatic arthritis; RANKL, receptor activator of nuclear factor-κB ligand; SLF, synovium-like fibroblasts; TNFα, tumour necrosis factor alpha; VEGF, vascular endothelial growth factor

In conclusion, the MBDA panel has been shown to be a reliable predictor of joint damage across clinical trials. It is tempting to speculate that the incorporation of additional serum proteins into this panel might increase its predictive power. Given the utility of a multi-biomarker panel demonstrated in RA it is also logical to suggest that such a panel might prove clinically useful in PsA. From this literature search it is evident that markers of angiogenesis and bone remodelling represent strong candidates. The predictive ability of such markers might be enhanced if incorporated into a panel with other newly identified molecules. It is interesting to note some markers have been identified exclusively in RA and PsA (Table 2). This identification suggests that distinct pathological mechanisms underpin joint destruction in these disorders. However, this might in part be a consequence of the lag in PsA research. The identification of additional biomarkers, particularly in PsA, should not only provide us with valuable prognostic information but also with a greater mechanistic insight into disease processes. Finally, identifying markers exclusive to one disease condition would also facilitate the development of diagnostic assays.

We suggest that the development of MRM assays for the candidate proteins identified here could be usefully developed to support the evaluation of multiplexed protein signatures that could predict joint damage. These assays could be developed into clinical diagnostic assays and used routinely in a daily practice and this should have a significant positive impact on patient care.

## Abbreviations

ACPA: Anti-citrullinated protein antibodies
A-SAA: Acute phase serum amyloid A
CCL: Chemokine ligand
COMP: Cartilage oligomatrix protein
CRP: C-reactive protein
CTX: C-terminal telopeptide of collagen
CXCL: Chemokine (C-X-C) motif ligand
ELISA: Enzyme-linked immunosorbent assay
ESR: Erythrocyte sedimentation rate
IL: Interleukin
MBDA: Multi-biomarker disease activity
MMP: Matrix metalloproteinase
MRM: Multiple reaction monitoring
PsA: Psoriatic arthritis
RA: Rheumatoid arthritis
RF: Rheumatoid factor
VEGF: Vascular endothelial growth factor
